# The drivers of global news spreading patterns

**DOI:** 10.1038/s41598-024-52076-6

**Published:** 2024-01-17

**Authors:** Shayan Alipour, Niccolò Di Marco, Michele Avalle, Gabriele Etta, Matteo Cinelli, Walter Quattrociocchi

**Affiliations:** 1 Sapienza University of Rome, Rome, Italy; 2 University of Florence, Florence, Italy

**Keywords:** Computational science, Complex networks

## Abstract

The web radically changed the dissemination of information and the global spread of news. In this study, we aim to reconstruct the connectivity patterns within nations shaping news propagation globally in 2022. We do this by analyzing a dataset of unprecedented size, containing 140 million news articles from 183 countries and related to 37,802 domains in the GDELT database. Unlike previous research, we focus on the sequential mention of events across various countries, thus incorporating a temporal dimension into the analysis of news dissemination networks. Our results show a significant imbalance in online news spreading. We identify news superspreaders forming a tightly interconnected rich club, exerting significant influence on the global news agenda. To further investigate the mechanisms underlying news dissemination and the shaping of global public opinion, we model countries’ interactions using a gravity model, incorporating economic, geographical, and cultural factors. Consistent with previous studies, we find that countries’ GDP is one of the main drivers to shape the worldwide news agenda.

## Introduction

The rapid evolution of the web has profoundly transformed the global dissemination of news. Nowadays, news flows incessantly, with media outlets promptly reporting unfolding events, and anyone can access this real-time information ecosystem. Furthermore, the Internet has intensified competition among news providers^1,2^ as they strive to capture users’ attention, contending with both traditional media and online news sources^3^. Advancements in data science have unlocked new possibilities for analyzing vast amounts of news content^4,5^ with extensive research efforts devoted to studying the impact of social media platforms on news dissemination^6–8^. A particular emphasis has been posed on their influence on misinformation^9,10^ and polarization^11–13^. In this dynamic environment, news spreads rapidly across the web, reaching numerous outlets in different countries and potentially shaping the news agendas of other nations^14,15^. Nevertheless, the reach and influence of the global news network vary significantly from one country to another. Factors such as culture, government censorship, language barriers, and digital division can impact news exchange between nations^16^. Therefore, gaining a comprehensive understanding of how news circulates on the Internet yields valuable insights into the economic and cultural influences among countries. It allows for characterizing news spreading routes, providing insights into the interconnected relationships and interdependence between different nations. The study of news spreading and the factors that influence the patterns of information and citation flow between countries have been topics of interest for several decades^17–20^. However, the dynamics of news dissemination across countries have evolved over time, especially with the rise of online media sources. In this landscape, some works emphasize the need for more comprehensive studies to capture these evolving trends^21^. Previous studies^22,23^ have shed light on the key factors influencing news dissemination, including economic strength, geographic location, and cultural elements. These factors are pivotal in shaping the pathways through which news flows across countries. Additionally, some other research^24–28^ have revealed hierarchical structures and clustering patterns that are driven by economic growth, language, and political freedoms. Such findings emphasize the complex dynamics in the global news network, where certain countries or regions may have stronger connections due to shared economic interests, linguistic affinities, or similar political environments.

However, previous research on news dissemination patterns faced limitations due to the challenges of obtaining extensive and diverse datasets that reflect the global media landscape. Recent breakthroughs in big data analytics and natural language processing have given rise to new tools that facilitate collecting and examining large volumes of news content. One such innovation is the Global Database of Events, Language, and Tone (GDELT) which automatically compiles and encodes news content from thousands of news sources across multiple languages^29,30^. In this study, we construct a comprehensive global network of news flows by leveraging GDELT data from 2022 to understand the peculiar characteristics of this network and what are the factors that determine its properties. Differently from the previous works in the field, our analysis focuses on a temporal approach^31^, i.e. the order in which news spreads within media outlets, thus moving beyond a simple examination of the frequency of countries’ names in news articles.

By studying the sequential mentions of events across countries, we aim to map the intricate interactions and reciprocal influences among nations in disseminating news. To achieve this result, our methodology involves identifying the initial source of each news event and tracing subsequent mentions of the event by other countries. By doing so, we can unravel the paths through which news travels across the global media landscape. This approach allows us to gain insights into how news events unfold, propagate, and are shared among countries, shedding light on the dynamics of news dissemination and the interconnectedness of nations in shaping the global information landscape. In addition to incorporating a temporal of news flow, we leverage a massive and unprecedented dataset of 140 million news articles from 37,802 domains across 183 countries sourced from GDELT. We find a significant skewness and inequality in the resulting graph, representing the interconnectedness of news dissemination. Specifically, we observe a small set of dominant countries that form a well-connected network, indicating their influential role in disseminating news globally and shaping the global public agenda. In pursuit of a deeper comprehension of the intricate dynamics underlying the patterns of news diffusion across countries, we use a gravity model. This methodological approach allows us to unravel the fundamental drivers of the observed skewed distribution. Our analysis reveals two primary determinants that significantly influence this phenomenon: the gross domestic product (GDP) and the geographic proximity of the countries involved. Notably, nations endowed with larger GDPs and closer geographical proximity demonstrate heightened interconnections and wield substantial influence within the global news network. Furthermore, we acknowledge the salient role common languages play, which actively shapes the flow of news across nations.

## Results

### News and countries

**Figure 1:**
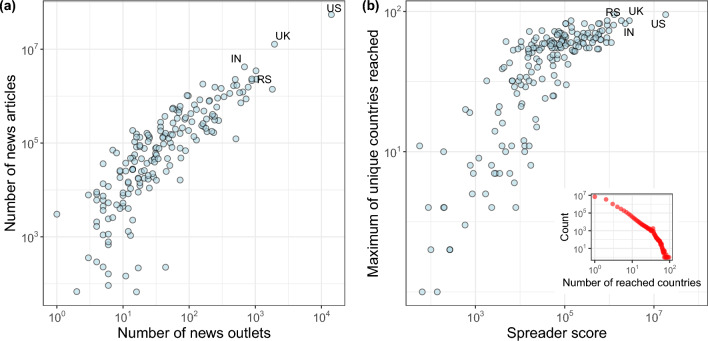
Left panel (**a**) describes the number of news outlets and published news articles for each country. Right panel (*b*) shows how many times a country served as the initial news spreader of the event and the maximum virality of events spread by each country. The inset in (**b**) reports the virality distribution (i.e. the number of unique countries an event gets mentioned in) for all events regardless of the initial spreader country.

To assess the extent of news outlet activity across different countries, we rely on the Source-Country dataset, as detailed in the Methods section, which allows us to identify and analyze news outlets alongside their respective news articles. To visualize the relationship between the number of news outlets and the corresponding quantity of news articles published by each country, we present Fig. 1a. This graphical representation depicts the interplay between these two variables and shows that countries with more news outlets tend to publish more news articles, following approximately a power law.

We define the initial spreader(s) as the country or countries that first mention the event. Since the GDELT dataset has a time granularity of 15 min, multiple countries can be identified as the initial spreaders for a single event. This is because two or more news outlets may have posted an article within the same 15 min window.

For each event, we consider the number of countries in which that event was mentioned. We call the distribution of these measures the *virality* distribution. Moreover, we define the *spreader score* for a country *i* as the number of times *i* belongs to the set of initial spreaders of an event. Figure 1b depicts the spreader score of a specific country plotted against the maximum number of countries reached by events published by that country.

Figure 1b indicates that the United States (US) emerged as the initial top spreader in 2022, significantly surpassing other countries. The United Kingdom (UK) and India (IN) followed closely behind. Moreover, we deduce that countries with higher spreader scores are more likely to have their events go viral at least once. The inset plot in Fig. 1 illustrates the overall distribution of virality, suggesting that most events tend to circulate within their countries.

### Structural analysis on news spreading

In this section, we aim to quantify the heterogeneity in the news-spreading process by means of a directed weighted network $G = (V, E)$ built using the GDELT dataset. The set of countries, denoted as *V*, represents the nodes in the network. A directed edge $(i, j) \in E$ is created if country *j* mentions an event successively after country *i*. The weight associated with each edge, denoted as $w_{ij}$, represents the number of times this sequential mentioning occurs. Please refer to the Methods section for more detailed information on this construction. The resulting network consists of 183 countries and 23,033 edges.

**Figure 2:**
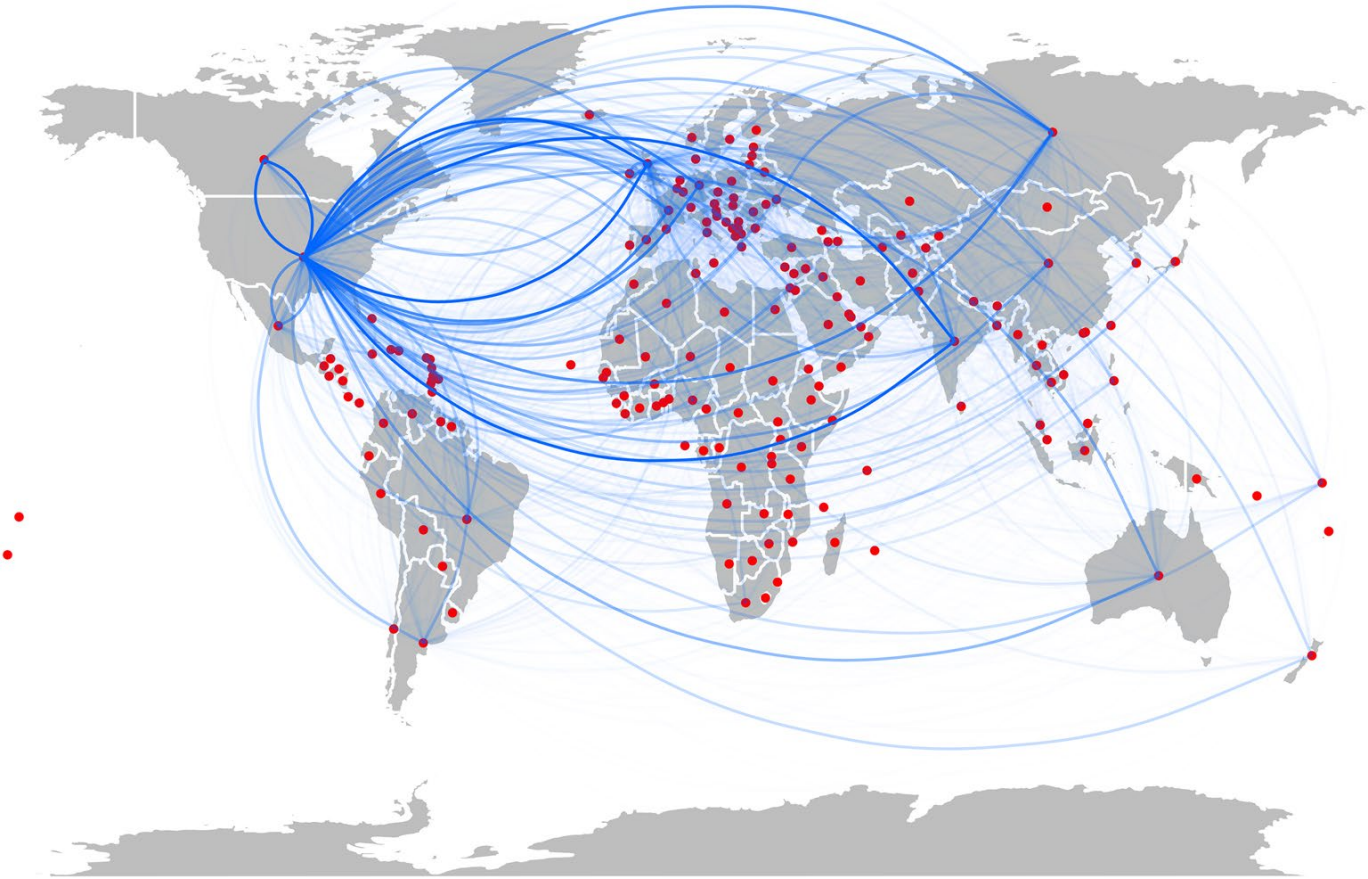
A graphical representation of the network obtained using GDELT data. The transparency of the link is proportional to the inverse of its weight.

**Figure 3:**
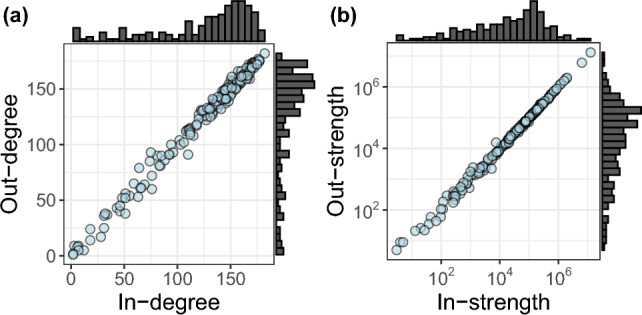
(**a**) relation between in and out-degree and (**b**) relation between in and out-strength. In both cases, they are highly correlated.

The network’s density, which indicates the proportion of existing connections compared to all possible connections, is 0.69. This value suggests that relatively few pairs of countries lack an exchange of information. Figure 3 illustrates the relationship between in-degree and out-degree (i.e., the number of in and out links attached to each node), as well as in-strength and out-strength (i.e., the sum of weights of in and out links attached to each node) within the network.

The degree distributions, both in-degree, and out-degree, reveal that most countries in the network have many connections. This indicates that countries tend to be mentioned by many other countries (in-degree) or mention many other countries (out-degree) at least once in the news-spreading process. Consistently with the high network density, the distribution on degrees suggests that countries are actively involved in disseminating news and have significant interactions with other countries.

The in-strength and out-strength distributions exhibit a similar pattern but on a logarithmic scale. This indicates that while most countries have a relatively high level of involvement in receiving or transmitting information, a few stand out with exceptionally high levels of strength in news dissemination. These countries play a prominent role in spreading the news, indicating a concentration of influence in the network.

An interesting observation is a high correlation between the in-degree and out-degree ($\rho = 0.99, p < 2.2 \times 10^{-16}$). This can be justified by the high reciprocity (i.e. proportion of links in both directions) exhibited by the network, with a value of 0.91. This surprisingly high reciprocity, together with high network density, indicates that subsequent mentions are usually mutual (i.e., both countries may mention an event in any order) and that connections among certain countries are more likely to be absent than unreciprocated. This suggests that some events are never mentioned in certain countries. Furthermore, when comparing the weights of mutual edges, they tend to have similar values. This implies that the extent of news diffusion between countries is well-balanced, as indicated by the similarity in the weights of reciprocal edges. Please refer to Supplementary Figure 1 in supplementary information for additional details and visualizations.

To examine the organizational principles of the network, we analyze its topology concerning the associated weights. In line with previous studies^32^, we compare the unweighted and weighted clustering coefficients to gain insights into the correlation between weights and network topology. Specifically, we define $C_w (s)$ as the weighted directed clustering coefficient^33^ and *C*(*s*) as its unweighted counterpart, both averaged over nodes with a strength of *s* (see Methods for details about the coefficients). Both quantities aim at expanding the insight provided by the reciprocity measure, searching for triplets of nodes with reciprocated links that may be considered as the building blocks for highly cohesive groups of countries in the network of news flow.

**Figure 4:**
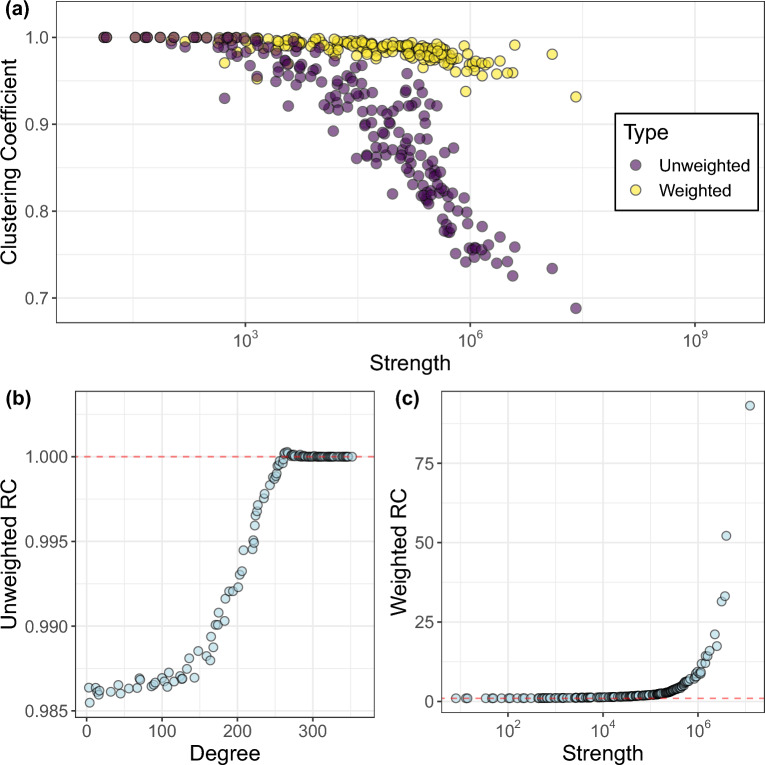
(**a**): Comparison between unweighted and weighted clustering coefficient. The weighted one takes higher values for higher strength. (**b**): Normalized rich-club (RC) coefficient for each degree value. (**c**): Normalized weighted rich-club coefficient for each strength value.

Figure 4(**a**) shows the results of the comparison of $C_w (s)$ and *C*(*s*) as the strength increases.

The weighted directed clustering coefficient $C_w (s)$ is approximately equal to the unweighted clustering coefficient *C*(*s*) for low-strength nodes. However, $C_w (s)$ takes on higher values for high-strength nodes than *C*(*s*). This finding suggests that while higher-strength nodes tend to form fewer triangles in terms of network topology, the triangles they do form are more likely to consist of edges with higher weights. In other words, there is a core group of countries where information flows preferentially, contributing to higher weighted clustering coefficients. This indicates the presence of strong interconnectedness and concentrated information exchange within this core group of countries. This is also suggested by Supplementary Figure 2 in supplementary information. Moreover, to further validate the results, we compare the two distributions of clustering coefficients (weighted and unweighted) with that of two appropriate null models. The results show that only the weighted clustering coefficient distribution differs from the null model, suggesting the importance of the weighted structure of the network. The interested reader can find additional details in supplementary information.

The observed scenario in terms of clustering coefficient and the observed in and out-strength distributions form the basis for detecting a rich-club phenomenon^34^. A rich-club refers to a group of prominent and tightly interconnected nodes that exert control over the flow of information in the network^32,35–37^. Figure 4 compares the normalized unweighted and weighted rich-club coefficients (refer to the Methods section for further details). The normalized rich-club coefficient measures the extent to which high-degree nodes tend to be more interconnected with each other compared to what would be expected by chance taking into account an appropriate null model of network rewiring. Consistent with the previous findings, we observe that only a strong weighted rich-club ordering (with the coefficient significantly higher than 1 for high-degree nodes) is evident, confirming our earlier hypothesis. Specifically, the eight largest countries (according to their strength) within the rich-club are the United States (US), the United Kingdom (UK), Canada (CA), India (IN), Russia (RS), Germany (GM), France (FR), and Ukraine (UP). These countries play a prominent role in the weighted network, forming a core where information flow is significantly concentrated and interconnected. Contrary to expectations, it is noteworthy that Ukraine belongs to the rich-club. This is likely due to the extensive coverage of events concerning the war between Russia and Ukraine in 2022, that contributed to its central position in the network.

**Figure 5:**
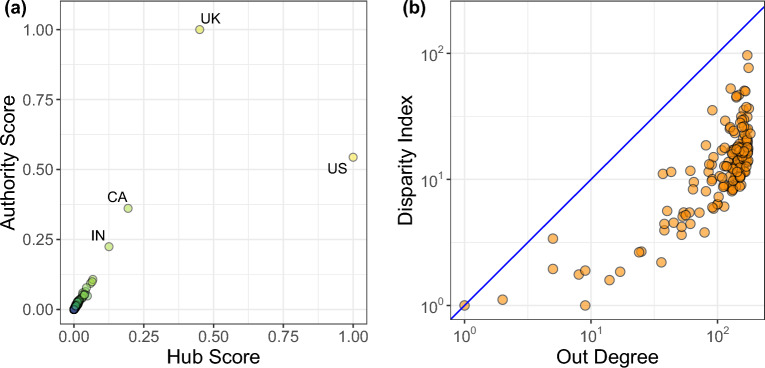
(**a**) Comparison between (weighted) Hub and Authority scores, nodes are color-coded according to their strength. We observe that US dominates as a Hub of the Network, while UK is the biggest Authority. (**b**) Disparity index $\gamma _i (k)$ versus the out degree of network’ nodes. We observe an increasing value of $\gamma _i (k)$ when the out Degree is greater.

All of the previous analyses indicate that only a small set of countries is actively involved in news diffusion, while the participation of other countries is relatively minimal. However, it is also interesting to examine the individual role of each country in the network. To accomplish this, we employ the HITS algorithm^38^ and compare the Authority and Hub scores of each node in Fig. 5, using the weights of the edges to represent the intensity of interaction. A node can be considered an Authority if it is linked by many Hubs, and, accordingly to this recursive procedure, a node can be considered a Hub if it points towards many Authorities. In our specific case, a Hub is a node that has many relevant out links and that is likely to be a source of news that are relevant for other prominent countries; an Authority, instead, is a country which immediately follows a Hub in the news flow and can be considered as a country for which events shared by Hubs are likely to be of high relevance.

The plot confirms that the United States (US), the United Kingdom (UK), Canada (CA), and India (IN) have a significant influence on news diffusion.

Specifically, US and UK emerge as the most influential countries of the network. The first has a Hub score close to one, indicating that it tends to be the source of many links, i.e. many countries follow them. On the other hand, the latter shows an Authority score close to one, indicating that it tends to follow many links, witnessing its importance in spreading information. Their relationship, with respect to this measure, reflects its interpretation as previously mentioned. In particular, US identifies itself as a news initiator that is promptly covered by the UK, which is likely to cover also other news by relevant countries such as Canada and India. Another noteworthy effect, related to the roles of US and UK, is their almost symmetrical relationship (which does not emerge in random instances of the model as shown in supplementary information) in the Hub and Authority diagram which may be considered an indicator of both an unbalanced, yet mutual, relationship and of a more general relevance in the interaction with other countries which underlines how these two countries play a central role in disseminating news on a global scale.

### Heterogeneity of diffusion and gravity model

In the previous section, we demonstrated that only a small group of countries actively participate in the dissemination of information, indicating a high degree of heterogeneity in how news is spread. To further quantify this aspect, we proceed by computing transition probabilities associated with each edge., i.e.

1 $$\begin{aligned} p_{ij} = \frac{w_{ij}}{\sum _{k = 1}^{deg(i)} w_{ik}}. \end{aligned}$$

The transition probability $p_{ij}$ represents the proportion or percentage of information produced by country *i* that flows to the country *j*. We are primarily interested in understanding if the observed behavior could emerge randomly. In Fig. 5b, we calculate the disparity index $\gamma _i (k)$^39^ (see Methods) and display it. The blue line indicates the maximum value that $\gamma _i (k)$ can assume. The values observed in Fig. 5b indicate that news diffusion heterogeneity increases with the node’s out-degree. This suggests that news diffusion does not occur randomly or uniformly across the network. If random, we would expect a more consistent behavior, resulting in $\gamma _i (k) \approx 1$ for any given *k*. The increasing values of $\gamma _i (k)$ concerning the out-degree imply that certain countries play a more influential role in news diffusion than others. This further result supports, from a slightly different perspective, the notion that news spreading is characterized by heterogeneity, where some countries have a more significant impact and influence in disseminating information than others.

We propose using a gravity model (GM) applied explicitly to news dissemination to investigate the factors influencing the observed behaviour in news diffusion. This approach has been employed in some prior studies, albeit with slight variations in network considerations^28^. For our analysis, we employ the CEPII gravity dataset^40^, which provides comprehensive economic data on international trade and various aspects of the global economy. However, since this dataset is updated only until 2020, we merge it with GDP data from 2022 obtained from the International Monetary Fund (IMF)^41^. Doing so, we aim to incorporate relevant economic factors into the gravity model for news diffusion. This approach will allow us to examine how economic variables, such as GDP, influence news flow between countries.

Our considered model is:

2 $$\begin{aligned} logit(p_{ij}) = \beta _0 + \beta _1 log(GDP_i) + \beta _2 log(GDP_j) + \beta _3 log(d_{ij}) + \beta _4 C_{ij} + \beta _5 L_{ij} + \beta _6 S_{ij}, \end{aligned}$$

where:

1. $GDP_i$ is the GDP of country *i* measured in billions of dollars;
2. $d_{ij}$ is the distance between the most populated cities of the two countries *i* and *j*, measured in *km*;
3. $C_{ij}$ is a dummy variable equal to 1 when country *i* and *j* share a common border;
4. $L_{ij}$ is a dummy variable equal to 1 when country *i* and *j* share a common language;
5. $S_{ij}$ is a dummy variable equal to 1 when country *i* and *j* share a common language spoken by at least $9\%$ of the population;

Note that our dependent variables are the logit of $p_{ij}$, namely

3 $$\begin{aligned} logit(p_{ij}) = \log \left( \frac{p_{ij}}{1-p_{ij}}\right) , \end{aligned}$$

i.e. the log odds of *i* continuing the flow in country *j*. Considering the specified model, coefficients themselves might not have direct and intuitive interpretations in the original scale. In fact, a change in an independent variable induces a change in the logit-transformed dependent variable and, if exponentially transformed, in its odds (i.e., the ratio in the probability of news flow happening between countries *i* and *j* to the probability of it not occurring). For example, a unit change in $GDP_i$ changes the value of $\frac{p_{ij}}{1-p_{ij}}$ of a quantity $e^{\beta _1}$ and similarly for the other logged independent variables.

To ensure the assumptions of our regression procedure are satisfied and to account for the potential correlation of errors within countries or country pairs, we employ Ordinary Least Squares (OLS) with robust clustered standard errors. Although estimating the parameter $\beta _k$, where $k \in {1, \ldots , 6}$, using standard OLS is common, it may underestimate the actual variance of the parameters^42^. This underestimation is due to the potential correlation of errors within the country, such as the correlation between country pairs. To address this issue, we use OLS with robust clustered standard errors. This involves specifying a clustering variable (in our case $d_{ij}$) that independently identifies each country pair, regardless of the direction. By employing robust clustered standard errors, we can ensure that all assumptions are satisfied and obtain more reliable estimates of the parameters.

After merging the CEPII and IMF datasets, we obtain a network of 172 nodes and 20468 edges. Table 1 shows the estimated parameters’ values.

**Table 1:** Result of the GM fitting.

	Estimate	Std. Error	t value	Pr( $|$t|)	CI Lower	CI Upper
(Intercept)	-5.40	0.13	-40.34	$<0.01$	$-$ 5.66	$-$ 5.14
$\log (GDP_i)$	$-$ 0.13	0.01	$-$ 23.02	$<0.01$	$-$ 0.14	$-$ 0.12
$\log (GDP_j)$	0.78	0.01	148.60	$<0.01$	0.77	0.79
$\log (d_{ij})$	$-$ 0.54	0.01	-37.85	$<0.01$	$-$ 0.57	$-$ 0.51
$C_{ij}$	0.49	0.08	5.73	$<0.01$	0.32	0.65
$L_{ij}$	0.48	0.06	8.27	$<0.01$	0.37	0.60
$S_{ij}$	0.42	0.06	7.56	$<0.01$	0.31	0.53

The obtained results indicate that our model explains approximately 59% of the variance in the dependent variable ($R^2 = 0.59$).

The findings suggest that news flow tends to occur more frequently between countries that are geographically closer to each other. However, the coefficient for distance in the news diffusion model (approximately $-$ 0.54) is lower in absolute value compared to what is typically observed in real trade networks (which is approximately 1). This difference can be attributed to the fact that news diffusion does not have the same tangible cost associated with it as in trade. News can travel more easily and quickly across long distances without incurring significant transportation or logistical expenses. Thus, the impact of geographical distance on news diffusion is somewhat attenuated compared to its impact on trade flows.

Additionally, the results indicate that economic power plays a role in news diffusion. Countries with higher GDP are more likely to be the target of the news flow, while they are less likely to be the source of the flow, although this effect is relatively weak ($\beta _1 \approx -0.13$).

Regarding cultural factors, the study reveals that the presence of a shared language has a substantial influence on the transmission of news. This is substantiated by the positive and statistically significant coefficients assigned to the language variables ($L_{ij}$, $S_{ij}$) in the model.

In summary, the analysis indicates that several factors, including geographic proximity, economic power, and cultural aspects like language, play a pivotal role in shaping the dynamics of news diffusion among nations. These findings emphasize the significance of considering these factors when examining the patterns of information flow on an international scale.

## Conclusion

In this paper we analyzed the network of global news diffusion in 2022. Our results depict a highly unbalanced situation in how news flow worldwide, with a rich-club of countries that act as superspreaders. Interestingly, we find that the countries’ *GDP* is one of the major factors that establish the role of countries in the news diffusion setting.

Our results were obtained using a network-based analysis and a gravity model to investigate the organizational structure and primary drivers of news dissemination in the digital realm. Moreover, in contrast to prior research, our study incorporates a temporal dimension into the analysis of news diffusion networks and uses a huge dataset provided by GDELT.

We highlight that possible limitations of our work are linked to the model of diffusion we used. In fact, an edge (*i*, *j*) between two countries does not represent in all cases a direct flow between country *i* and *j*, i.e. we are not sure that *j* mentions the event because *i* did. Moreover, the Multilingual Source-Country dataset provides only an approximation of the geographic country of origin of news outlets.

However, since we use a large amount of data and a time-aggregating approach, it is reasonable to suppose that the network provides a good approximation of how news flows between pairs of countries. This is also justifiable by the observed network heterogeneity and the departure of the network structure from random models (as shown in supplementary information).

Although our study uses a different dataset and modelling approach, and despite potential data limitations related to the ongoing Russia-Ukraine conflict, our findings align with prior research, shedding also new light on the dynamics that occur in the news diffusion environment.

## Methods

### Data collection and preprocessing

In our study, we explored global news dynamics by leveraging the power of big data to understand the trajectory and network structure of news articles. Our primary data sources include the GDELT 2.0 dataset and the Multilingual Source-Country dataset. These datasets offer a comprehensive view of news articles in multiple languages, enabling us to conduct a granular analysis of both Western and non-Western media narratives. The following sections detail our data collection methodology and the preprocessing steps we took.

#### GDELT dataset

We used the GDELT 2.0 to collect online news articles from January 1 to December 31, 2022, with a 15-minute update resolution. The Global Database of Events, Language, and Tone (GDELT) is an independent, non-profit project providing a vast, updated global news and events database in different languages. The dataset covers news articles written in English and 65 other languages, allowing us to delve deep into non-Western media. The data in GDELT 2.0 is organized into three main tables: Events, Mentions, and the Global Knowledge Graph (GKG). The Events table lists events with related general information. The Mentions table instead contains each news article referring to an event from the Events table. Finally, the GKG table provides detailed information about each article’s actors, emotions, and themes. We used the Mentions table to track a story’s trajectory and network structure as it flowed through the global media system from country to country. In the Mentions table, each mention (i.e. news article) of an event is given its entry in the dataset. This means an event mentioned in 100 news articles will be listed 100 times in the Mentions table. The dataset resulting from this collection, described in Table 2, consists of 140 million news articles referencing 51 million unique events. To ensure data accuracy, we excluded news articles from domains not present in the Source-Country dataset, resulting in 125 million news articles and their respective country of origin. We explain GDELT’s event identification process in the supplementary information.

**Table 2:** Dataset breakdown.

	Records (M)
English articles	99.5
Non-english articles	40.5
Total articles	140
Articles with domains inside source-country	125

#### Multilingual source-country dataset

This dataset (gdelt-bq.extra.sourcesbycountry) estimates the country of origin for major online news outlets monitored by GDELT, using the primary geographic focus of the outlet over the monitored time frame. GDELT’s documentation mentions challenges related to outlets with insufficient coverage volume or geographic emphasis and regional wire services with bureaus in particular countries^43^. Despite the challenges, the dataset is considered a reasonable approximation of the geographic country of origin for these news outlets and is available on GDELT’s website.

### Network construction

Starting from our collected data, for each event *k* we consider a directed weighted graph $G_k = (V_k,E_k)$. In particular, $V_k$ is the unique set of countries that mention *k*, while $(i,j) \in E_k$ if country *j* mentions *k* successively after country *i*. In more detail, consider two successive times $t_i, t_{i+1}$ (we have $t_{i+1}-t_i \ge 15$ min) and let $C_k(t_i),C_k(t_{i+1})$ be the set of countries that mention event *k* at time $t,t+1$ (respectively). If $T_k$ is the lifespan of event *k*, we have that $E_k = \bigcup _{i = 1}^{T_k-1} C_k(t_i)\times C_k(t_{i+1})$. Since it is possible to have multiple edges, we associate to each of them a weight $w_{i,j}^k$ that represents their multiplicity. This procedure results in a collection of graphs $G_k$, one for each event.

We obtain a final network $G = (V,E)$ defined as

$$\begin{aligned} G = \bigcup _{k} G_k \end{aligned}$$

and $w_{ij} = \sum _k w_{ij}^k$, i.e. we overlap all the networks associated with each event. Thus, *G* is a directed weighted network in which an edge between (*i*, *j*) means that *j* mentions an event successively after *i* a number of times equal to $w_{ij}$. Such network construction is akin to what is called time-aggregated weighted network in temporal network theory^44^. To focus on the relationship among countries, we delete all the loops, i.e. edges that start and end in the same country, obtaining a simple graph.

To clarify the previous steps, we provide an example that describes how we construct the network for a single event $\epsilon _0$. Consider the following list of countries mentioning $\epsilon _0$ (by means of at least one of their news outlets).

**Table:** 

Country	Event	Time stamp
*US*	$\epsilon _{0}$	$t_{0}$
*CA*	$\epsilon _{0}$	$t_{1}$
*US*	$\epsilon _0$	$t_2$
*IT*	$\epsilon _0$	$t_3$
*UK*	$\epsilon _0$	$t_4$
*US*	$\epsilon _0$	$t_5$
*CA*	$\epsilon _0$	$t_6$

This table can be turned into a chain of (direct) mentions, i.e.

$$\begin{aligned} US \rightarrow CA \rightarrow US \rightarrow IT \rightarrow UK \rightarrow US \rightarrow CA. \end{aligned}$$

Therefore, we obtain the following edges

$$\begin{aligned} E = \{(US,CA),(CA,US),(US,IT),(IT,UK),(UK,US),(US,CA)\}. \end{aligned}$$

In this simple example, all edges have a weight equal to 1, except for (*US*, *CA*) which appears twice and therefore has a weight equal to 2. Such edges can be used to build the graph $G_0$ related to the event $\epsilon _0$.

### Measures on networks

#### Weighted clustering coefficient

Clustering coefficients are widely used in the network science literature, with the aim of studying the interconnectedness of nodes’ neighbours. However, fewer measures have been proposed for weighted directed networks.

Since one of our aims is to study the organization of news diffusion network, we decided to consider the Clustering Coefficient proposed by Clemente and Grassi^33^, which can be defined as follows

4 $$\begin{aligned} C_i = \frac{0.5 \left[ (\textbf{W}+\textbf{W}^T)(\textbf{A}+\textbf{A}^T)^2\right] _{ii}}{s_i \left( d_i -1\right) - 2s_i^{\leftrightarrow }} \end{aligned}$$

where $\textbf{A},\textbf{W}$ are the unweighted and weighted adjacency matrices of the network, $s_i$ is the strength of node *i* and $s_i ^{\leftrightarrow }$ is defined as the strength of bilateral arcs, i.e.

$$\begin{aligned} s_i ^\leftrightarrow = \sum _{i \ne j} a_{ij} a_{ji} \frac{w_{ij} + w_{ji}}{2}. \end{aligned}$$

The numerator of (4) takes into account all directed triangles that node *i* actually forms with its neighbours, weighted with the average weight of the links connecting a node *i* to its adjacent *j* and *k*. The denominator counts the total number of directed triangles that it could form, taking weights properly. In our context, comparing unweighted and weighted clustering coefficients sheds light on the relations between the network topology and its weights. In such a way, we are able to quantify possible inequalities in how countries spread news.

#### Rich-club

Rich-club refers to a network subgraph made up of the most prominent nodes (from a topological or non-topological point of view) being highly interconnected with each other. Many studies report that these nodes tend to be important for the overall structure and function of the network^35,45–47^. In more detail, given a weighted graph $G = (V,E)$ with binary adjacency matrix *A* and weighted adjacency matrix *W* ($W_{i,j} \in \left( 0, +\infty \right) )$, the topological (i.e. unweighted) measure that allows to detect a rich-club with respect to nodes of degree *k* is:

5 $$\begin{aligned} \phi (k) = \frac{2 m_{>k}}{n_{>k} (n_{>k} -1)}, \end{aligned}$$

where $m_{>k}$ and $n_{>k}$ are the number of links and nodes of the subgraph $G_{>k} \subseteq G$ inducted by nodes with degree higher than *k*.

To consider weighted networks, the presence of a rich-club must be detected with respect to a certain richness parameter *r* that allows ranking the nodes with respect to it. Common examples of richness parameters can be degree, strength or other measures of centrality^36,37^.

Given such a measure, it is possible to define the weighted rich-club as:

6 $$\begin{aligned} \phi ^w (r) = \frac{W_{>r}}{\sum _{i = 1}^{E_{>r}} W_i^{rank}} \end{aligned}$$

where $w_i ^{rank} \ge w_{i+1} ^{rank}$, with $i = 1,2,\ldots , |E|$ are the weights of the links ranked in increasing order. Thus, (6) measures the fraction of weights shared by the richest nodes compared to the total amount they could share using the strongest links of the network.

However, the two definitions alone, don’t assure the existence of a rich-club effect. To measure that, a suitable null model that preserves some properties of the original graph must be considered^48^. In this context, we are interested in maintaining the strength distribution, therefore we consider a set of randomized networks obtained from *G* by reshuffling locally the weights of the outgoing links, as proposed by Opsahl et al.^36^.

We define $\phi _{null} ^{w} (r)$ as the value of (6) computed for the random null model. Thus, we detect a rich-club effect by computing the ratio

7 $$\begin{aligned} \rho ^w (r) = \frac{\phi ^w (r)}{{\bar{\phi }}_{null}^w (r)}, \end{aligned}$$

where ${\bar{\phi }}_{null}^w (r)$ is the mean across *N* generated null model. A value of $\rho ^w (r) > 1$ indicates that rich nodes concentrate their flow towards other rich nodes more than expected in a set (usually containing 100 randomised instances) of null models. Note that (7) can be applied with no problem to the case of unweighted networks. However, in this case, we consider a null model in which the degree sequence is preserved, using a rewiring algorithm^49^. In the context of news diffusion networks, a rich-club is a subset of the countries in which information flows preferentially and more frequently.

#### HITS algorithm

HITS (Hyperlink-Induced Topic Search)^38^ is a web page ranking algorithm used to evaluate web pages relevance based using the network structure, defined by a directed graph $G = (V,E)$. The basic idea is to consider a web page to be important if it is linked to other important pages, considering two types of scores associated with each page: *Hub* and *Authority*. In particular, a page with a high Hub score points to many good Authorities and, vice-versa, a high Authority score indicates that a page points to many good Hubs. Moreover, weights of the network can be used as connection strengths. In our context, a Hub is a country that tends to be the source of the news time mentions, i.e. many (Authority) countries follow it in the diffusion. On the other hand, an Authority is a country that tends to be the target of the news time mentions, i.e. it follows many (Hub) countries in the news diffusion and assumes a central role in how information is disseminated.

To give a mathematical description of the two measures, let’s denote with *x*(*p*) (*y*(*p*)) the Authority score (Hub score) of page *p*. The algorithm initially sets $x(p) = y(p) = 1$ and then updates the values according to the following simple rules:

1. For each *p*, $$x(p) = \sum _{\{q: (q,p) \in E\}} y(q)$$;
2. For each *p*, $$y(p) = \sum _{\{q: (p,q) \in E\}} x(q)$$;
3. Normalize the Authority scores such that $\sum _{p \in V} x(p)^2 = 1$;
4. Normalize the Hub scores such that $\sum _{p \in V} y(p)^2 = 1$;

Linear Algebra tools assure that the previous algorithm converges to fixed points $\bar{x}$ and $\bar{y}$. Moreover, experimental evidence indicates that this convergence is obtained with a limited number of steps.

#### Disparity index

The disparity index has been widely used in many fields like economics^50^, ecology^51^, physics^52^ and network science^39,53^. It measures the inhomogeneities in the weights at the local level of nodes and it is defined, for a node *i* of degree *k*, as

8 $$\begin{aligned} \gamma _i (k) = k \sum _{j} p_{ij} ^ 2, \end{aligned}$$

where $p_{ij}$ are the transition probabilities associated with each edge, as defined in (1). Its value ranges between 1 (perfect homogeneity, i.e. all the links have the same weight) and *k* (perfect heterogeneity, i.e. only a link carries the whole node strength). In the news diffusion landscape, (8) can be used to understand if the behaviour observed in the network occurs randomly or not. In fact, in the first case, we could expect $\gamma _i \approx 1$, i.e. a uniform behaviour.

### Supplementary Information

Supplementary Information.

## Data Availability

The datasets generated and/or analysed during the current study are available in the GDELT repository, https://blog.gdeltproject.org/gdelt-2-0-our-global-world-in-realtime/.
