# Supplementary material for: The drivers of global news spreading patterns
Source: Sci Rep. 2024 Jan 17;14:1519. doi: 10.1038/s41598-024-52076-6 (PMC10794245; doi:10.1038/s41598-024-52076-6)
Supplement: Supplementary file 1 — Supplementary Information. [file 41598_2024_52076_MOESM1_ESM.pdf]

The Drivers of Global News Spreading Patterns - Supplementary Information

Shayan Alipour[1], Niccolò Di Marco[2], Michele Avalle[1], Gabriele Etta[1], Matteo Cinelli[1], and Walter Quattrociocchi[1,*]

[1]Sapienza University of Rome, Italy
[2]University of Florence, Italy
[*]walter.quattrociocchi@uniroma1.it

Supplementary Information

GDELT event identification process

GDELT identifies events by monitoring news reports and using natural language processing (NLP) techniques to extract quotes, people, organizations, and locations mentioned in the text. The number of unique events in GDELT's dataset, 51 million, may appear unusual at first glance. To clarify this, we provide an example based on GDELT's documentation. Suppose we have a short text: "The United States criticized Russia yesterday for deploying its troops in Crimea, in which a recent clash with its soldiers left ten civilians injured." In this case, GDELT would generate three distinct events: "US CRITICIZES RUSSIA," "RUSSIA TROOP-DEPLOY UKRAINE (CRIMEA)," and "RUSSIA MATERIAL-CONFLICT CIVILIANS (CRIMEA)." This method allows GDELT to capture multiple events within a single news article, leading to a higher number of unique events in our dataset.

Interactions between mutual edges

Consider the directed weighted network of news diffusion G. To understand if mutual edges share the same amount of information, we normalize the weights of mutual edges of G using

$$p_{ij} = \frac{w_{ij}}{\sum_{k=1}^{deg(i)} w_{ik}}, \tag{1}$$

i.e. we compute the transition probabilities between countries. Therefore, if $w_{ij} \approx w_{ji}$ there is evidence that the flow of information is similar between source and target (and vice-versa).
Supplementary Figure 1 shows that in general, we can deduce that connected countries do not differ much in how they share information, especially for high weights links.

Core of countries

To try to understand how countries are organised in the network, we consider a notion of distance between nodes using the idea that "close" countries are the ones that have more interactions (i.e. a high weight). Therefore, we update the weights of G considering:

$$d_{ij} = \frac{1}{w_{ij}} \tag{2}$$

obviously, this does not correspond to a distance in a mathematical sense (for example, $d_{ij} \neq d_{ji}$ in general), but this transformation allows us to compute distances taking into account the closeness of countries in terms of news diffusion.
Supplementary Figure 2 shows a heatmap of the shortest distances computed with the weights $\mathbf{d}$. The heatmap order followed the out-strength value.

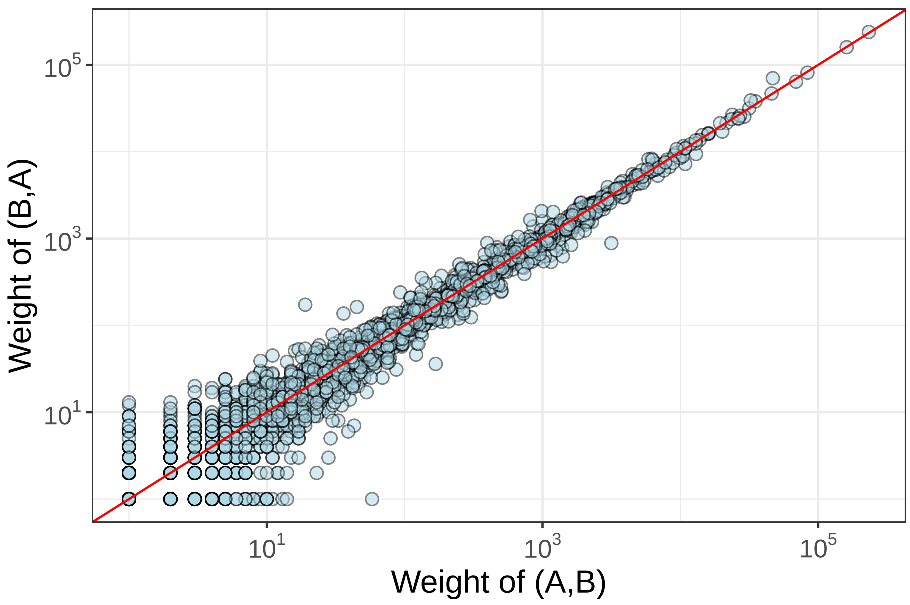

Supplementary Figure 1: Comparison between weights of mutual edges. Only a sample of 2000 points is considered for better graphics reasons.

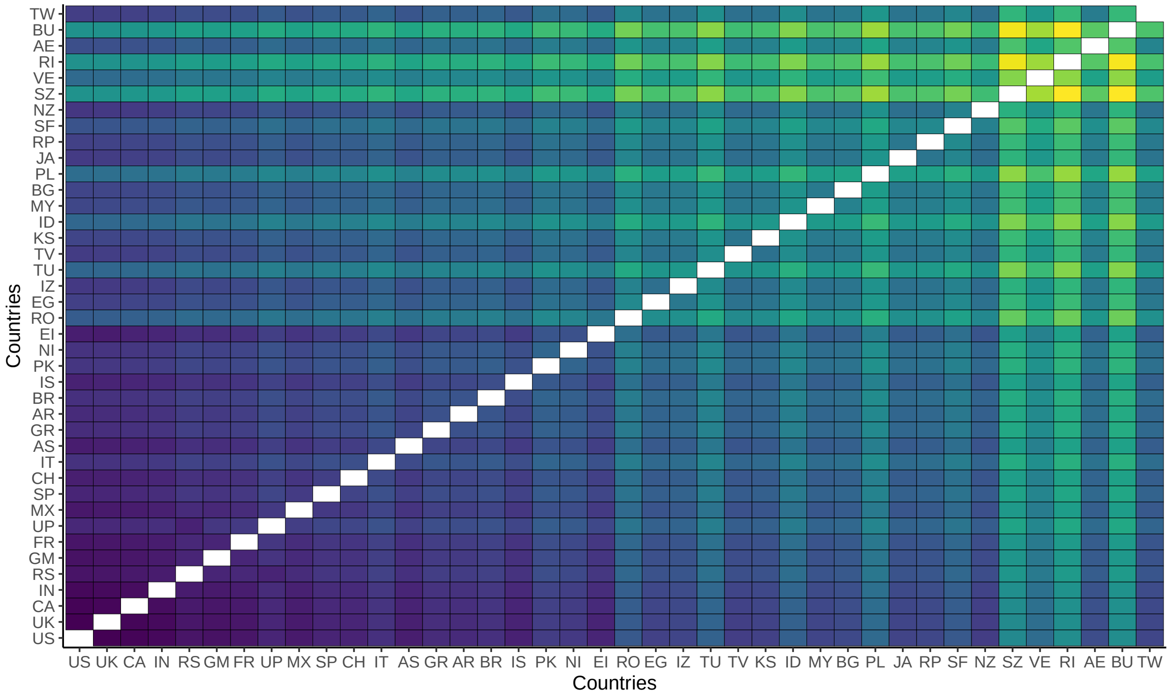

Supplementary Figure 2: Heatmap of shortest paths among 40 countries with the highest out-strength. We use (2) as weights. The countries are ordered using out-strength.

Interestingly, countries with high out-strength tend to have low distances in the network. This suggests the existence of a core of countries in which information flows preferentially.

Validation of measures

To further validate our results, we consider two different null models depending on whether the considered measure is weighted or not, accordingly with the case of the rich-club coefficient. We recall that, in the first case, we use a standard rewiring algorithm that reconnects the endpoint of network edges chosen uniformly at random. In the second case, we shuffle the weights of the out-edges of each node, thus preserving the strength of each vertex.

Clustering coefficient

For the unweighted clustering coefficient, we first create a randomized network applying $100|E|$ rewiring steps. Then, we compare its clustering coefficient distribution with that of the real network, using a permutation test ran with $N = 10^4$ permutations. We obtain $p = 0.94$, suggesting that the real network and the null model share the same distribution. On the other hand, for the weighted clustering coefficient, we create a randomized network by shuffling its out weights and then we compare the original distribution with that of this network. In this case, the permutation test outputs $p < 0.001$, confirming that the distributions are different. Since the edge density is 0.69 and the peculiar characteristics of the network depend more on their weighted structure than on the links' disposition (as suggested in the main paper), it is expected to obtain the same distribution for the unweighted clustering coefficient. On the other hand, we observe higher values for the weighted clustering coefficient than expected in the null model, indicating that the triangles are formed by bigger weights than expected randomly.

HITS

We use the same (weighted) null model to highlight possible differences arising from running HITS on a randomized network. To do that, we created 100 different randomized networks and we averaged their Hubs and Authorities score. The results are showed in Supplementary Figure 3.

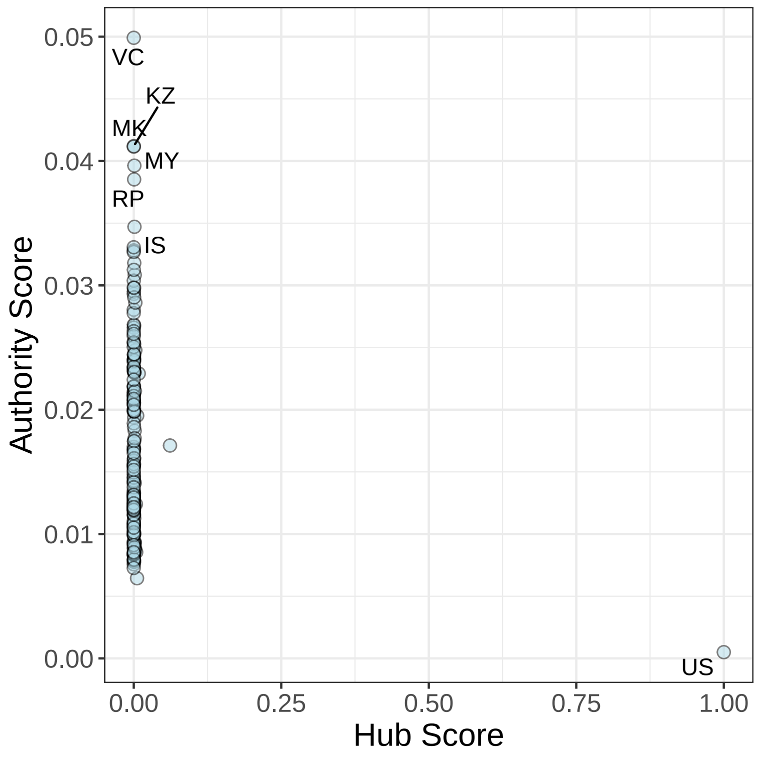

Supplementary Figure 3: Results of Hits algorithm on the network in which out-weights are randomly shuffled.

Interestingly, HITS still classifies US as the biggest Hub but it totally loses its role as Authority (i.e.

the fact that US is followed is more expected, but following US is not random). Interestingly, all the other nodes have a marginal role in the null model, differently from what is observed in the real network.
